# Health-related quality of life and its demographic, clinical and psychosocial determinants among male patients with hypertension in a Ghanaian tertiary hospital

**DOI:** 10.4314/gmj.v56i1.2

**Published:** 2022-03

**Authors:** Vincent Boima, Alberta K Yeboah, Irene A Kretchy, Augustina Koduah, Kofi Agyabeng, Ernest Yorke

**Affiliations:** 1 University of Ghana Medical School, Department of Medicine and Therapeutics, College of Health Sciences, University of Ghana, Box 4236 Accra, Ghana; 2 School of Pharmacy, Department of Pharmacy Practice and Clinical Pharmacy, College of Health Sciences, University of Ghana, P. O. Box LG 43, Legon, Ghana; 5 National Inspectorate Board. Ministry of Education, PMB M18, Accra, Ghana

**Keywords:** Distress, Health-Related Quality of Life, Hypertension, Insomnia, Males, Medication adherence

## Abstract

**Objectives:**

This study aimed to evaluate Health-related quality of life (HRQoL) among male patients with hypertension and its associated demographic, clinical and psychosocial factors.

**Design:**

This was a facility-based cross-sectional study

**Setting:**

This study was carried out at the outpatient department in Korle-Bu Teaching Hospital

**Participants:**

Three hundred and fifty-eight hypertensive patients were recruited for this study

**Data collection:**

Information on socio-demographic characteristics, clinical features, insomnia, medication adherence, psychological distress, sexual dysfunction and HRQoL were obtained through patient-reported measures using structured questionnaires and standardised instruments.

**Statistical analysis/Main outcome measure:**

The study assessed HRQoL among male hypertensive patients. One-way ANOVA was used to compare the average scores of the various domains of HRQL across the independent variables. Multivariate linear regression models with robust standard errors were used to determine factors associated with quality of life.

**Results:**

Participants with poor perceived overall HRQoL was 14.0%. Comparatively, HRQoL (mean ± SD) was the least in the physical health domain (56.77±14.33) but the highest in the psychological domain (58.7 ± 16.0). Multivariate linear regression showed that income level, educational level, insomnia, overall satisfaction, sexual desire and medication adherence were significant predictors of HRQoL. Average scores of HRQoL domains reduced with a higher level of sexual desire dysfunction.

**Conclusion:**

HRQoL among male hypertensive patients was negatively affected by insomnia, sexual desire dysfunction, educational level and adherence to antihypertensive medications but positively affected by income level. Clinical practice and policy processes should be directed at these factors to improve HRQoL.

**Funding:**

No external funding

## Introduction

Hypertension continues to be a global public health concern and a major risk factor for cardiovascular diseases.^1^,^2^ Being the most common cardiovascular problem, the prevention and control of hypertension remain a major public health issue of high importance. This is because about 9.4 million deaths occur annually worldwide due to hypertension and its associated complications.^3^

The World Health Organization (WHO) estimates the worldwide prevalence of hypertension to be about 1.13 billion people. This figure is projected to increase to 1.56 billion by 2025.^4^ However, two-thirds of the affected persons live in low- and middle-income countries, with an overall prevalence of 32.3% estimated from a systematic review of previous studies.^5^–^7^ Compared with the WHO Region of the Americas, which has the least hypertension prevalence of 18%, the African region has been reported to have the highest prevalence of 27%.^7^

In Ghana, previous studies have reported a prevalence of 19% to 48%^8^ and indications from the 2017 demographic and health survey showed a prevalence of 11.4% to 30.4% for hypertension.^9^ A review of worldwide trends shows that the prevalence of hypertension in males is higher than in females, with the WHO reporting that in 2015, 1 in 4 men compared with 1 in 5 women had hypertension.^7^ While studies demonstrate that men are more prone to developing hypertension before age 50, women tend to have an increased risk post menopause.^10^, ^11^. With sex differences in the aetiology and effect of hypertension, men also tend to experience more blood pressure burden on their organs^11^ although a review of some studies found no differences between men and women in terms of the effect of elevated blood pressure on cardiovascular disease outcomes.^12^

In sub-Saharan Africa, blood pressure control is generally poor among persons with hypertension,n even with evidence to support the fact that hypertension is on the rise.^13^, ^14^ Efforts to improve blood pressure control, reduce the risk of complications and improve health-related quality of life (HRQoL) are considered necessary due to the fact that patients with hypertension have been reported to have poor HRQoL compared with healthy individuals.^15^ HRQoL is an important aspect in the evaluation of the well-being of patients with hypertension and attempts at improving this factor becomes a major goal in hypertension management.^16^ The concept of HRQoL refers to the extent to which a person's well-being and function relates with his/her perceptions of physical, mental, and social domains of life.^17^ HRQoL is mostly affected by health issues which are associated with the disease burden and reflects the extent of coping and health-related outcomes of patients.^18^ For patients with hypertension, their HRQoL tends to be influenced by demographic characteristics, antihypertensive medications and adherence^19^, physical and mental health co-morbidities, sexual dysfunction^20^ and sleep disorders.^19^

Since hypertension and its control is a common problem and men are prone to have a greater blood pressure burden on their organs, an assessment of HRQoL, therefore, becomes an important indicator in evaluating treatment outcomes and well-being in men affected by hypertension.

## Methods

### Study design and Participants

The study was a hospital-based cross-sectional study at the Korle-Bu Teaching Hospital in Accra, Ghana, between January 2017 and April 2017. The hospital is a 2000 bed capacity facility with 21 clinical and diagnostic departments and has an average outpatient attendance of 1,500. The study was conducted among 358 hypertensive male patients. Sample size calculation details have previously been reported for as part of the broader study on biopsychosocial determinants of hypertension management in males.^30^ The participants were contacted by the research team in the specialist, medical and general out-patient clinics of the Department of Medicine.inclusion criteria for this study included being hypertensive male on medications for at least 12 months, and age 18 years and over. Eighteen to twenty-five patients were consecutively recruited per day in the order in which they presented during each clinic visit. Hypertension was defined as a Systolic Blood Pressure (SBP) of ≥ 140mmHg and Diastolic Blood Pressure (DBP) of ≥ 90mmHg and when participants were on any hypertensive treatment Blood pressure was measured using an Omron electronic BP machine, (HEM-907-E7), made in Japan and they are inspected regularly (biannually) by the hospital technical unit in Korle-Bu Teaching Hospital. Blood pressure measurements were taken after 5 minutes rest, in the dominant arm of seated patients on three occasions at two minutes intervals. The average of the last 2 readings was recorded.

### Data collection

The participants who met the inclusion criteria and consented to participate were recruited into the study consecutively. An interviewer-administered questionnaire approach was used. The study participants completed questionnaires regarding socio-demographic characteristics, HRQoL, sexual dysfunction, psychological distress, and sleep problems.

### WHO Quality Of Life Scale (Whoqol-Bref)

The WHOQOL-BREF self-report instrument consists of 26- items which is a cross-cultural shorter version of the WHO quality of life scale. There are domains evaluated which are physical, psychological, social relationships and environmental health^21^ with five Likert-type response options with higher scores indicating better quality of life. The overall perceived HRQoL item was dichotomised as poor (combination of 1- very poor, and 2-poor) and good (3-neither poor nor good, combination of 4- good and 5-very good) HRQoL. The internal consistency checks (Cronbach alpha) for the various domains were: Psychological – 0.73, Social relationships – 0.65, Environment – 0.83 and Physical Health – 0.71.

### International Index for Erectile Function (IIEF)

This self-report instrument consists of 15 items assessing sexual dysfunction in the following domains: erectile function (6-items), orgasmic function (2-items), sexual desire (2-items), intercourse satisfaction (3-items) and overall satisfaction dysfunction (2-items) rated on a 6-point scale(0–5)^31^. each domain score generated by computing total score for the items in each domain and lower scores indicating high dysfunction.^31^

In this study, the IIEF scale was reliable with Cronbach alpha score of 0.959.

### Kessler Psychological Distress Scale (KPDS)

The KPD scale measured distress (symptoms of anxiety and depression) in the most recent 4-week period. It consists of 10 items with response options from 1 (none of the time) to 5 (all of the time). The total scores range from 10 to 50, with scores < 20, 20–24, 25–29 and ≥30 representing mild, moderate, and severe distress. 32 This scale was reliable, with a Cronbach alpha value of 0.886 in this study.

### Athens Insomnia scale (AIS)

The measurement of insomnia in this study was based on the AIS which assesses eight factors with response options on a scale of 0 to 3.^33^ The first five factors relate to nocturnal sleep and the last three factors identify daytime dysfunction. Cumulative score of all factors was reported as the sleep outcome. A cut-off score ≥6 was used to indicate insomnia.^33^, ^34^ The scale's reliability coefficient had a Cronbach alpha of 0.834 in this study.

### Medication Adherence Questionnaire (MAQ)

This self-report instrument consists of 4 items with ‘yes’ or ‘no’ response options on past medication use.^35^ The scores are categorised as high (0), medium (1–2) and low adherence (3–4) when participants answered ‘no’ to all the questions, ‘yes’ to one question, or ‘yes’ to two or more questions, respectively. In this study the reliability coefficient based on the Cronbach alpha was 0.701.

### Data Analysis

Data were entered into SPSS version 22 and later exported into STATA version 15 for analysis. Frequencies and percentages were used to summarise categorical variables, while means and standard deviations were used in summarising continuous variables. Normality tests for continuous variables and the residuals of the various linear regression models were done with skewness and kurtosis test and a histogram with a normal distribution curve superimposed on it.

Average scores with standard deviations were reported for the HRQoL domains, while the frequencies and percentages were reported for the perceived overall quality of life and general health. One-way ANOVA and Welch's t-test compared average scores across the independent variables. Multivariate linear regression models with robust standard errors were used to determine factors associated with quality of life. All statistical tests were done at 5% significance level.

### Ethics approval

The study was approved by the by the Institutional Review Board at the Noguchi Memorial Research Institute for Medical Research, University of Ghana, Legon (reference number 035-16/17) and was carried out in accordance with ethical standards involving human participants. Only patients who gave informed consent participated in the study. All participants provided written informed consent.

## Results

### Patient characteristics

The average ± SD age of the study participants was 56.2 ± 13.5 years (range: 25 – 91years). More than half of the study participants had lived with hypertension for at least five years (52.5%). Half of the participants were prescribed an average of two medications for their condition (Table 1).

**Table 1:** Characteristics of male hypertensive patients receiving treatment and Comparison of mean HRQoL scores across characteristics of male hypertensive patients receiving treatment at the KBTH

	Frequency	Psychological		Social Relationships		Environment		Physical Health	
	
	n(%)	Mean ± SD	p-value	Mean ± SD	p-value	Mean ± SD	p-value	Mean ± SD	p-value
Overall: Mean ± SD		**58.68 ± 16.01**		**58.04 ± 19.95**		**57.44 ± 16.80**		**56.77 ± 14.33**	

**Income (GHC)**			<0.001		0.012		<0.001		0.008
Below 500	75(20.95)	58.49 ± 15.73		57.11 ± 19.89		57.01 ± 16.55		56.16 ± 14.66	
500–999	155(43.3)	57.25 ± 15.08		55.15 ± 19.87		54.12 ± 15.25		55.48 ± 13.54	
1000–2999	103(28.77)	57.89 ± 16.67		60.77 ± 18.81		59.13 ± 17.29		56.91 ± 13.88	
≥3000	25(6.98)	71.32 ± 15.11		67.48 ± 21.95		72.40 ± 16.51		65.96 ± 17.26	
**Marital status**			<0.001		0.003		0.001		0.009
Single	28(7.82)	63.07 ± 17.12		63.86 ± 19.39		62.71 ± 18.50		59.11 ± 12.98	
Married	253(70.67)	60.47 ± 15.71		59.49 ± 19.12		58.70 ± 16.29		57.99 ± 14.61	
Divorced	56(15.64)	50.75 ± 12.64		49.89 ± 20.36		52.71 ± 13.89		52.04 ± 13.23	
Widowed	21(5.87)	52.38 ± 18.59		54.48 ± 24.06		47.81 ± 21.93		51.52 ± 12.16	
**Length of Diagnosis**			0.010		0.158		0.014		0.023
< 2 Years	19(5.31)	63.19 ± 17.92		61.57 ± 19.29		60.82 ± 16.98		61.14 ± 14.42	
2 – 4 Years	49(13.69)	55.82 ± 14.18		57.54 ± 17.77		56.36 ± 16.01		55.78 ± 14.14	
5 – 7 Years	119(33.24)	57.47 ± 15.49		59.10 ± 21.82		54.56 ± 16.25		56.66 ± 12.9	
8 – 10 Years	171(47.77)	55.19 ± 14.61		51.50 ± 14.47		53.14 ± 16.35		52.03 ± 12.92	
> 10 Years	19(5.31)	61.59 ± 16.67		57.03 ± 23.10		61.69 ± 17.63		56.17 ± 16.19	
**Education**			0.045		0.05		0.3		0.225
Basic	19(5.31)	60.82 ± 14.41		51.24 ± 14.97		60.39 ± 13.76		55.37 ± 14.69	
Secondary	49(13.69)	59.27 ± 15.26		60.70 ± 18.18		56.40 ± 15.62		57.03 ± 13.37	
Tertiary	119(33.24)	56.78 ± 16.50		58.08 ± 22.00		56.80 ± 18.39		56.28 ± 14.66	
Others	171(47.77)	66.53 ± 17.86		58.47 ± 19.57		62.11 ± 15.44		63.05 ± 15.70	
**Age**	56.20 ± 13.50	0.03§	0.513	-0.14 §	0.010	0.03 §	0.623	-0.13 §	0.014
**Number of Medications**	2.00 (2.00, 4.00)	0.09 °	0.094	0.02 °	0.763	0.10 °	0.062	0.06 °	0.225
**Sleeping Hours**	7.00 (6.00, 8.00)	0.07 °	0.165	0.01 °	0.906	0.04 °	0.415	0.06 °	0.243
**Clinical Disorders**									
**Insomnia**	7.64 ± 4.40		<0.001		<0.001		<0.001		<0.001
No	130(36.31)	66.54 ± 15.95		64.76 ± 21.04		64.86 ± 16.69		64.05 ± 14.23	
Yes	228(63.69)	54.19 ± 14.25		54.20 ± 18.27		53.21 ± 15.36		52.61 ± 12.66	
**Erectile function**	**12.49 ± 8.56**		<0.001		<0.001		<0.001		<0.001
Mild		74.74 ± 12.42		75.61 ± 18.05		73.68 ± 16.82		71.52 ± 13.14	
No dysfunction		60.66 ± 15.56		67.91 ± 19.41		61.74 ± 16.30		62.17 ± 11.64	
Mild to medium dysfunction		55.42 ± 15.14		57.52 ± 16.07		54.83 ± 14.63		56.71 ± 11.92	
Medium dysfunction		51.35 ± 9.89		50.54 ± 16.62		48.75 ± 12.77		48.99 ± 12.13	
Severe dysfunction		61.41 ± 17.62		51.91 ± 21.58		58.87 ± 17.51		54.14 ± 15.63	
**Orgasmic function**	**3.47 ± 2.30**		<0.001		<0.001		0.001		<0.001
Severe		69.12 ± 10.01		74.96 ± 12.38		66.72 ± 14.18		68.92 ± 11.59	
Mild dysfunction		59.57 ± 17.05		62.69 ± 18.65		59.54 ± 17.24		60.39 ± 11.82	
Mild to medium dysfunction		54.61 ± 13.98		55.24 ± 19.66		53.58 ± 15.62		53.22 ± 14.44	
Medium dysfunction		59.73 ± 16.85		51.92 ± 19.86		57.21 ± 17.11		53.80 ± 14.94	
**Sexual Desire**	**4.39 ± 2.11**		<0.001		<0.001		<0.001		<0.001
Mild dysfunction		70.21 ± 13.84		72.98 ± 18.57		68.12 ± 15.46		65.77 ± 12.99	
Mild to medium dysfunction		58.50 ± 15.67		61.9 ± 18.00		57.62 ± 16.22		60.42 ± 12.47	
Medium dysfunction		57.05 ± 14.18		53.29 ± 17.98		55.93 ± 14.11		52.44 ± 13.32	
Severe dysfunction		53.45 ± 17.89		45.3 ± 19.55		51.70 ± 20.40		48.34 ± 15.02	
**Intercourse Satisfaction**	**6.34 ± 4.39**		<0.001		<0.001		<0.001		<0.001
No dysfunction		70.17 ± 16.26		77.31 ± 17.23		69.74 ± 17.83		70.69 ± 12.86	
Mild dysfunction		62.25 ± 16.77		63.25 ± 20.25		61.00 ± 17.44		61.45 ± 14.28	
Mild to medium dysfunction		52.40 ± 14.35		56.70 ± 17.00		53.44 ± 16.53		54.04 ± 11.73	
Medium dysfunction		56.26 ± 12.55		55.95 ± 18.84		53.24 ± 14.42		53.68 ± 13.66	
Severe dysfunction		60.37 ± 16.69		51.63 ± 19.55		58.26 ± 15.75		54.47 ± 14.29	
**Overall Satisfaction**	**3.75 ± 1.93**		0.077		<0.001		0.027		0.001
No dysfunction		75.00 ± 8.49		75.00 ± 1.00		44.00 ± 8.49		59.50 ± 4.95	
Mild dysfunction		64.41 ± 12.14		75.86 ± 13.01		66.18 ± 14.43		63.45 ± 8.95	
Mild to medium dysfunction		59.49 ± 16.97		62.27 ± 18.59		59.09 ± 17.94		59.79 ± 14.50	
Medium dysfunction		56.07 ± 14.47		56.46 ± 18.13		55.06 ± 15.12		55.78 ± 13.04	
Severe dysfunction		59.19 ± 16.97		50.78 ± 21.30		56.67 ± 17.22		52.92 ± 15.50	
**Psychological distress**	**21.60 ± 7.75**		<0.001		0.256		<0.001		<0.001
No mental disorder		65.03 ± 17.61		59.98 ± 21.69		61.81 ± 18.49		62.06 ± 15.13	
Mild mental disorder		55.65 ± 12.94		55.76 ± 17.16		55.23 ± 14.31		52.81 ± 14.42	
Medium mental disorder		50.56 ± 11.11		55.33 ± 16.94		50.57 ± 12.14		51.28 ± 9.91	
Severe mental disorder		55.38 ± 13.62		59.37 ± 21.90		57.15 ± 16.98		54.40 ± 11.55	
**Medication Adherence**	2.07 ± 1.43		<0.001		<0.001		0.008		<0.001
Low	151(42.18)	65.32 ± 15.00		61.85 ± 20.08		61.85 ± 20.08		64.52 ± 15.03	
Medium	143(39.94)	55.90 ± 14.65		55.16 ± 19.38		55.16 ± 19.38		53.90 ± 15.33	
High	64(17.88)	49.20 ± 14.81		55.47 ± 19.70		55.47 ± 19.70		48.66 ± 17.44	

SD: Standard deviation, LQ: Lower quartile, UQ: Upper quartile, GHC: Ghana Cedis

§ Pearson's correlation values

° spearman rank correlation values

**Figure 1:**
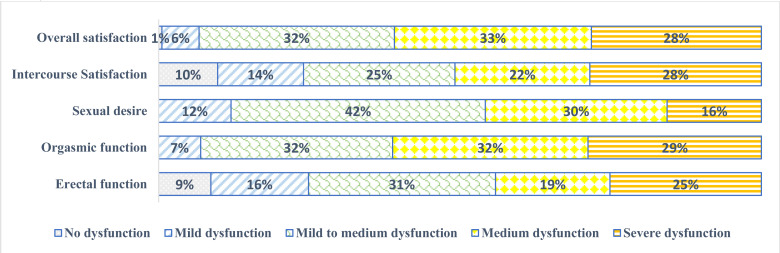
Distribution of sexual dysfunction among male hypertensive patients receiving treatment at the KBTH

All the study participants experienced some level of orgasmic and sexual desire dysfunctions (Figure 1), 63.7% experienced insomnia, 56.4% (202/358) experienced psychological distress and 42.8%, 39.9% and 17.9% reported low, moderate, and high medication adherence levels (Table 1 and Fig. 1).

### HRQoL

In assessing the HRQoL among the study participants, the psychological domain recorded the highest mean ± SD subscale score of 58.7 ± 16.0, followed by social relationships (mean ± SD: 58.0 ± 20.0). The mean ± SD subscale score for the environment domain was 57.4 ± 16.8, while the physical health domain had the least subscale score (56.77±14.33).

Table 2 shows the multivariate linear regression model in determining the effects of the covariates on the various HRQoL domains. From the models, insomnia and Medication Adherence were significant predictors of all the four domains of HRQoL (p-value<0.001) In comparing the various HRQoL domain scores across the background characteristics of the study respondents using one-way-ANOVA test, participants who earned GHC 3000 (about US$ 703) and above, had significantly highest average scores compared to those in the lower income categories (p-value <0.05) for all four domains. In relation to marital status, the highest average scores were realised significantly among single participants compared with all the other groups (p-value <0.05). Newly diagnosed patients (< 2 years) recorded the highest average scores across all the various HRQoL domains except for the social domain (Table 2).

**Table 2:** Multiple linear regression results for factors associated with HRQoL scores of male hypertensive patients receiving treatment at the KBTH

	Psychological		Social relationships		Environment		Physical Health	
	
	β (95% CI)	P-value	β (95% CI)	P-value	β (95% CI)	P-value	β (95% CI)	P-value
**Income (GHC)**		**0.045**		0.432		**0.013**		0.242
*Below 500*	0		0		0		0	
*500–999*	2.64(-1.38, 6.67)		-1.64(-6.97, 3.70)		1.42(-2.84, 5.68)		1.98(-1.82, 5.79)	
*1000–2999*	1.25(-3.13, 5.64)		2.07(-3.58, 7.71)		3.75(-1.10, 8.60)		1.29(-2.81, 5.38)	
*≥3000*	9.48(2.57, 16.38)		6.99(-2.15, 16.12)		11.10(4.00, 18.20)		6.56(-0.01, 13.13)	
**Marital status**		**0.042**		0.432		0.263		0.972
*Single*	0		0		0		0	
*Married*	-4.54(-10.29, 1.20)		-1.92(-9.67, 5.83)		-5.13(-12.11, 1.85)		-0.84(-5.57, 3.89)	
*Divorced*	-8.97(-15.63, -2.32)		-5.28(-14.93, 4.37)		-5.48(-13.61, 2.65)		-0.44(-6.45, 5.57)	
*Widowed*	-7.96(-17.17, 1.24)		3.29(-10.29, 16.86)		-11.62(-23.2, -0.04)		-0.05(-6.55, 6.45)	
**Education**		**0.040**		**0.041**		0.066		0.098
None	0		0		0		0	
Basic	0.76(-7.52, 9.04)		-1.77(-10.25, 6.7)		5.23(-1.99, 12.45)		-2.58(-9.26, 4.10)	
Secondary	-0.63(-8.20, 6.94)		5.30(-2.47, 13.06)		2.02(-5.09, 9.14)		-2.47(-8.18, 3.24)	
Tertiary	-4.57(-11.96, 2.81)		0.03(-7.67, 7.73)		-0.95(-7.90, 6.00)		-5.27(-10.77, 0.23)	
**Insomnia**		**<0.001**		**0.013**		**<0.001**		**<0.001**
*No*	0		0		0		0	
*Yes*	-6.49(-9.94, -3.05)		-5.88(-10.54, -1.22)		-6.74(-10.31, -3.18)		-6.91(-10.07, -3.75)	
**Erectile function**		0.145		0.654		0.086		0.103
*No dysfunction*	0		0		0		0	
*Mild dysfunction*	-7.16(-13.72, -0.59)		1.90(-6.87, 10.67)		-8.03(-16.78, 0.72)		-4.38(-10.61, 1.86)	
*Mild to moderate dysfunction*	-6.59(-14.02, 0.83)		-0.22(-9.93, 9.49)		-10.12(-19.59, -0.66)		-4.24(-11.05, 2.57)	
*Moderate dysfunction*	-8.91(-16.97, -0.85)		-3.32(-14.45, 7.82)		-13.79(-23.68, -3.89)		-9.04(-16.46, -1.62)	
*Severe dysfunction*	-4.19(-15.22, 6.84)		0.47(-16.9, 17.85)		-9.64(-21.78, 2.50)		-6.14(-15.96, 3.68)	
**Orgasmic** **function**		0.538		0.486		0.798		0.341
Mild dysfunction	0		0		0		0	
Mild to moderate dysfunction	-4.15(-9.89, 1.58)		-5.81(-13.15, 1.54)		-0.96(-8.35, 6.43)		-4.07(-8.98, 0.83)	
Moderate dysfunction	-3.60(-9.91, 2.70)		-4.82(-12.9, 3.26)		-1.43(-9.12, 6.27)		-4.84(-10.13, 0.44)	
*Severe dysfunction*	-4.66(-12.84, 3.53)		-5.16(-16.93, 6.6)		-4.68(-14.87, 5.51)		-4.30(-11.78, 3.18)	
**Sexual Desire**		**0.013**		**0.016**		0.128		**0.006**
*Mild dysfunction*	0		0		0		0	
*Mild to moderate dysfunction*	-5.34(-10.26, -0.43)		-3.72(-10.54, 3.11)		-4.30(-10.09, 1.50)		0.81(-4.10, 5.72)	
*Moderate dysfunction*	-6.31(-12.08, -0.54)		-5.38(-13.33, 2.56)		-3.88(-10.56, 2.79)		-2.95(-8.40, 2.49)	
*Severe dysfunction*	-10.86(-17.41, -4.32)		-13.18(-22.26, -4.1)		-8.63(-16.35, -0.91)		-8.06(-14.55, -1.57)	
**Intercourse** **Satisfaction**		0.547		0.213		0.524		0.168
*No dysfunction*	0		0		0		0	
*Mild dysfunction*	-0.74(-7.68, 6.20)		-10.66(-20.41, -0.92)		-2.46(-11.26, 6.35)		-5.62(-11.88, 0.64)	
*Mild to moderate dysfunction*	-2.87(-9.81, 4.08)		-10.11(-19.27, -0.95)		-1.08(-9.85, 7.68)		-6.5(-12.45, -0.56)	
*Moderate dysfunction*	-0.03(-7.23, 7.18)		-10.63(-20.2, -1.06)		-2.31(-11.42, 6.81)		-6.51(-12.87, -0.15)	
*Severe dysfunction*	0.77(-7.39, 8.94)		-12.08(-23.85, -0.31)		2.08(-8.08, 12.25)		-3.82(-10.96, 3.32)	
**Overall Satisfaction**		0.844		0.120		**<0.001**		0.279
*Mild dysfunction*	0		0		0		0	
*No dysfunction*	3.98(-5.32, 13.28)		12.04(-2.82, 26.9)		36.98(27.78, 46.19)		13.89(-1.27, 29.05)	
*Mild to moderate dysfunction*	1.61(-7.12, 10.35)		3.44(-9.89, 16.76)		32.98(25.27, 40.69)		14.08(-0.65, 28.82)	
*Moderate dysfunction*	1.03(-7.91, 9.97)		1.56(-12.17, 15.3)		32.52(24.51, 40.52)		13.73(-1.39, 28.84)	
*Severe dysfunction*	-0.64(-11.13, 9.85)		-2.36(-17.57, 12.85)		31.39(21.80, 40.98)		11.11(-4.54, 26.76)	
**Medication** **Adherence**		**<0.001**		**0.027**		**<0.001**		**0.007**
*Low*	0		0		0		0	
*Medium adherence*	-4.07(-7.54, -0.59)		-4.94(-9.73, -0.16)		-5.43(-9.08, -1.77)		-2.40(-5.65, 0.85)	
*High adherence*	-10.81(-15.21, -6.4)		-7.75(-13.59, -1.90)		-9.91(-14.93, -4.88)		-5.26(-8.59, -1.93)	

**β**: estimated effect of covariate, CI is confidence interval; GHC: Ghana Cedis

Participants with insomnia had about 6 unit points less in all the four domains of HRQoL scores than those without insomnia. For medication adherence level, participants with a medium and high level of adherence had significantly lower HRQoL scores across all four domains than those with low adherence (p-value <0.05). The HRQoL scores for sexual desire significantly reduced with a higher level of sexual desire dysfunction (p-value <0.05) across all domains of the HRQoL except the Environmental domain. There was a significant positive relationship between the Environmental domain scores and the Overall satisfaction scores. An increase in the level of overall satisfaction dysfunction was associated with over 30 unit points higher HRQoL score (Environmental domain). Marital status was predictive of the Psychological HRQoL scores. Married, divorced, and widowed participants had 4.5, 9.0 and 8.0 lower Psychological HRQoL scores than the single ones respectively (p-value =0.042). Table 2.

The percentage of participants with poor perceived overall HRQoL was 14.0% (50/358) (Table 4). In comparing the mean scores for the four HRQoL domains with the perceived overall quality of life, the Welch's t-test showed that patients with poor perceived overall HRQoL scored significantly lower across all the four domains compared to those with good overall perceived HRQoL [(Psychological: 43.98 ± 11.78 vs 61.06 ± 15.33, p<0.001), (Social relationships: 44.60 ± 18.64 vs 60.22 ± 19.32, p<0.001), (Environment: 41.26 ± 15.31 vs 60.07 ± 15.52, p<0.001), (Physical Health: 43.80 ± 12.03 vs 58.87 ± 13.56, p<0.001)] Table 3

**Table 3:** Comparison of perceived overall HRQoL and measured (actual) HRQoL of male hypertensive patients re-ceiving treatment at the KBTH

	Overall Quality of Life			
			
	Poor [N= 50 (14.0%)]	Good [N= 308 (86.0%)]		
			
Domains	Mean ± SD	Mean ± SD	t-test statistic	p-value
Psychological	43.98 ± 11.78	61.06 ± 15.33	-9.08	<0.001
Social relationships	44.60 ± 18.64	60.22 ± 19.32	-5.47	<0.001
Environment	41.26 ± 15.31	60.07 ± 15.52	-8.04	<0.001
Physical Health	43.80 ± 12.03	58.87 ± 13.56	-8.06	<0.001

p-values were obtained from Welch t-test. SD: Standard Deviation

## Discussion

This study was designed to evaluate HRQoL and its determinants among male patients with hypertension. The overall average scores for the four domains of HRQoL were more than half of the total HRQoL scores. Psychological domain had the highest score while physical domain had the least score. The results showed that, at least 1 out of every 10 participants had poor perceived overall quality of life. Generally, participants with poorly perceived overall HRQoL had significantly lower scores in all four domains than those who perceived good overall HRQoL. Factors significantly associated with HRQoL were income level, educational level, insomnia, overall satisfaction, sexual desire and medication adherence. Furthermore, participants with sexual desire dysfunction had lower HRQoL scores.

The HRQoL scores observed in this study are similar to observations in some population-based studies.^22^ A previous study reported low HRQoL in hypertensive populations.^23^ This is similar to our study findings, where more than 40% of patients with hypertension had poor HRQoL. The psychological domain had the highest score in our study, while the physical domain had the lowest score. Previous studies showed differing outcomes where the psychological domain was affected more than the physical domain.^24^, ^25^ In contrast, other studies showed that the physical domain was affected more than the other domains of HRQoL.^26^

The findings of poor HRQoL among male hypertensive patients may be as a result of associated side effects of medications such as sexual dysfunction and other factors including sleep disturbance, adherence with medication, level of education and socioeconomic indicators as shown in this current study where the above factors were significantly related to HRQoL.

In previous studies from South Korea and Parkistan, hypertensive patients from low income groups had worse HRQoL.^27^, ^28^ Xiao et al assessed perceived economic burden caused by hypertension and found that both males and females perceived that low economic burden relating to hypertension was associated with better HRQoL.^29^ In their study low income was associated with lower scores for vitality and mental health among male patients with hypertension.

The findings from the above studies agree with our study results among male hypertensives where participants with lower income had lower scores across all domains of the HRQoL. This suggests that hypertensive patients with low income may have difficulty with the financial burden posed by their ill-health, which may negatively impact their quality of life. In line with this, all stakeholders including national health insurance scheme and other governmental agencies should consider supporting these patients adequately, including providing insurance coverage for their health care needs for better health outcomes.^30^

As with our study, a review of previous studies found insomnia co-morbid with other medical conditions that negatively affected the quality of life outcomes.^31^ This is supported by another study where over 40% of hypertensive patients seen by general practitioners had poor sleep or insomnia.^32^ In a cross-sectional analysis of a medical outcomes study among patients with chronic illness including hypertension and diabetes mellitus, insomnia was associated with worsened HRQoL across all domains especially psychological health, vitality and general perception domains.^33^ A cross-sectional survey by Uchmanowicz et al among elderly hypertensive patients revealed that sleep problems have negative impact on HRQoL especially in the physical health domain.^34^ One way of improving the HRQoL of patients with chronic physical conditions like hypertension may be to address problems with their sleep. This will help to control their blood pressures sufficiently enough to prevent development of attendant complications such as cardiovascular disease. This is because several studies have revealed positive correlations between insomnia and elevated blood pressure among hypertensive patients.^35^

The relationship between medication adherence and HRQoL in various studies produced conflicting results. Our study demonstrated that hypertensive male patients with a medium to high adherence had significantly lower scores across all domains of HRQoL. This contrasts with a study among adults aged 65 years and above in a large community study which showed an association between low antihypertensive medication adherence and low HRQoL scores. In a study among geriatric hypertensive patients, no association was demonstrated between self-reported medication adherence and HRQoL.^36^ Again, another study among adults with hypertension aged 35 to 80 years showed a weak negative correlation between self-reported medication adherence and HRQoL.^37^ The mechanisms through which HRQoL affects adherence remain unknown. However, studies among patients with diabetes identified the following as antecedents to medication adherence; the belief that they are able carry out the behavior.^38^ attitudes and knowledge about disease treatment.^39^ perceived level of competence 38 and overall outlook on life.^40^ Nevertheless, future research is required to accurately explain the mechanisms through HRQoL affects adherence to medication.

Study participants in our study experienced various forms of sexual dysfunction which may have significantly lowered their scores in all four domains of HRQoL.

The consistency of our findings with the previous studies^41^, ^42^ provide further evidence supporting previous observations that the issue of sexual dysfunction in hypertensive patients is a major public health concern. Thus, timely recognition and appropriate intervention will be important so that the quality of life of male patients with hypertension and their partners are not compromised.

Although this study has provided vital information on the HRQoL and its associated factors in male patients with hypertension who tend to experience greater burden of blood pressure, the extent to which the findings are generalisable are limited due to the fact that this study concentrated only on men from a single health centre within the country. Another limitation of this study was the use of self-reported measures which may be prone to recall or social desirability bias. Despite the above limitations, the current study has substantial strengths for clinical practice and policy.

## Conclusion

HRQoL among male hypertensive patients was negatively affected by insomnia, sexual desire dysfunction, educational level and adherence to antihypertensive medications but positively affected by income level. It is important to note that the predictive factors of HRQoL among male hypertensive patients in this study are modifiable and should be considered in policy formulation for best clinical practice by health professionals in Ghana.
